# Triggering molecular assembly at the mesoscale for advanced Raman detection of proteins in liquid

**DOI:** 10.1038/s41598-018-19558-w

**Published:** 2018-01-18

**Authors:** Martina Banchelli, Marella de Angelis, Cristiano D’Andrea, Roberto Pini, Paolo Matteini

**Affiliations:** Institute of Applied Physics ‘Nello Carrara’, National Research Council (IFAC-CNR), via Madonna del Piano 10, Sesto Fiorentino, Italy

## Abstract

An advanced optofluidic system for protein detection based on Raman signal amplification *via* dewetting and molecular gathering within temporary mesoscale assemblies is presented. The evaporation of a microliter volume of protein solution deposited in a circular microwell precisely follows an outward-receding geometry. Herein the combination of liquid withdrawal with intermolecular interactions induces the formation of self-assembled molecular domains at the solid-liquid interface. Through proper control of the evaporation rate, amplitude of the assemblies and time for spectral collection at the liquid edge are extensively raised, resulting in a local enhancement and refinement of the Raman response, respectively. Further signal amplification is obtained by taking advantage of the intense local electromagnetic fields generated upon adding a plasmonic coating to the microwell. Major advantages of this optofluidic method lie in the obtainment of high-quality, high-sensitivity Raman spectra with detection limit down to sub-micromolar values. Peculiarly, the assembled proteins in the liquid edge region maintain their native-like state without displaying spectral changes usually occurring when dried drop deposits are considered.

## Introduction

Optofluidic devices mainly consist of an integration of a microfluidic process with a photonic system acting synergically to maximize activity and performance of chemical and biological analyses^1,2^. Droplet-based processes ruled by complex physical and chemical mechanisms and integrated within photonic architectures represent a particular type of optofluidic mycrosystems^3–5^. During evaporation of a solution droplet containing a dispersion of molecules on a solid surface a flow inside the droplet is induced causing a molecular flux toward the contact line at the solid/liquid interface. This initiates a typical mesoscale process in which the structural rearrangements of molecules across several length scales (from dispersed in solution to assembled at the solid/liquid interface) are achieved spontaneously under local non-equilibrium conditions^6,7^. Complex high-order molecular structures are generated during the process as a consequence of the interplay of competitive and cooperative forces without deterministic dependence on the starting conditions of the initial objects^8,9^. These dynamics typically pertain to living systems, which necessitate metastable structures of enough dynamic and adaptive nature that can effectively respond to external stimuli to generate biological processes. The interplay of solvent evaporation and solute interaction with the surface can eventually generate molecule deposition in dense macroscopic patterns upon complete drying of the solution, which are frequently referred as “coffee-ring” stains. These represent favourable regions for effective detection of analytes in dry conditions, which has long attracted a large interest from the scientific community and exploited in the context of so-called drop-coating deposition Raman methods^10–14^. However, drying of sessile droplets is actually a complex non-equilibrium process that may be not adequately controlled^15–18^. In recent literature the use of confined spaces to control kinetics and dynamics of evaporative dewetting of liquid volumes has been proposed^19–21^. Such control might confer the optofluidic system with potential for quantitative and reproducible measurements, as well as could be exploited for molecular enrichment in pre-determined locations such as on plasmonic structures to collect an enhanced photonic response.

In the present work we take inspiration from these concepts to setup a microfluidic system for advanced detection of biomolecules in wet conditions coupled with Raman analysis. Specifically the system is engineered to track the Raman modes of a protein solution during its evaporation when contained inside a microwell reservoir. An optimization of the system is obtained by equipping it with a temperature controller for slowing down the evaporation rate and by coating the bottom of the microwell with a plasmonic nanoparticle layer for additional enhancement of the Raman signal. Remarkably, the microfluidic system enables Raman detection at the edge of a biomolecule solution before its complete withdrawal, whereas the main part of the methods proposed so far are suited for detection in dry conditions, which is limiting, e.g., for the analysis of proteins in their native and functional state. On getting control over the evaporation-induced molecular flux, the formation of highly dense domains of proteins at liquid edges in their native conformation is favoured. Upon inspection of these crowded protein assemblies, an overall gain of ~10^5^ in the Raman signal is produced with respect to that from bulk solution when identical acquisition parameters are used. The proposed system paves the way toward advanced biochemical and biological investigations, as well as shows potential for ultrasensitive detection of biomolecules in their own environment.

## Results and Discussion

### Evaporation in a microwell and Raman enhancement at the liquid edge after meniscus rupture

**Figure 1:**
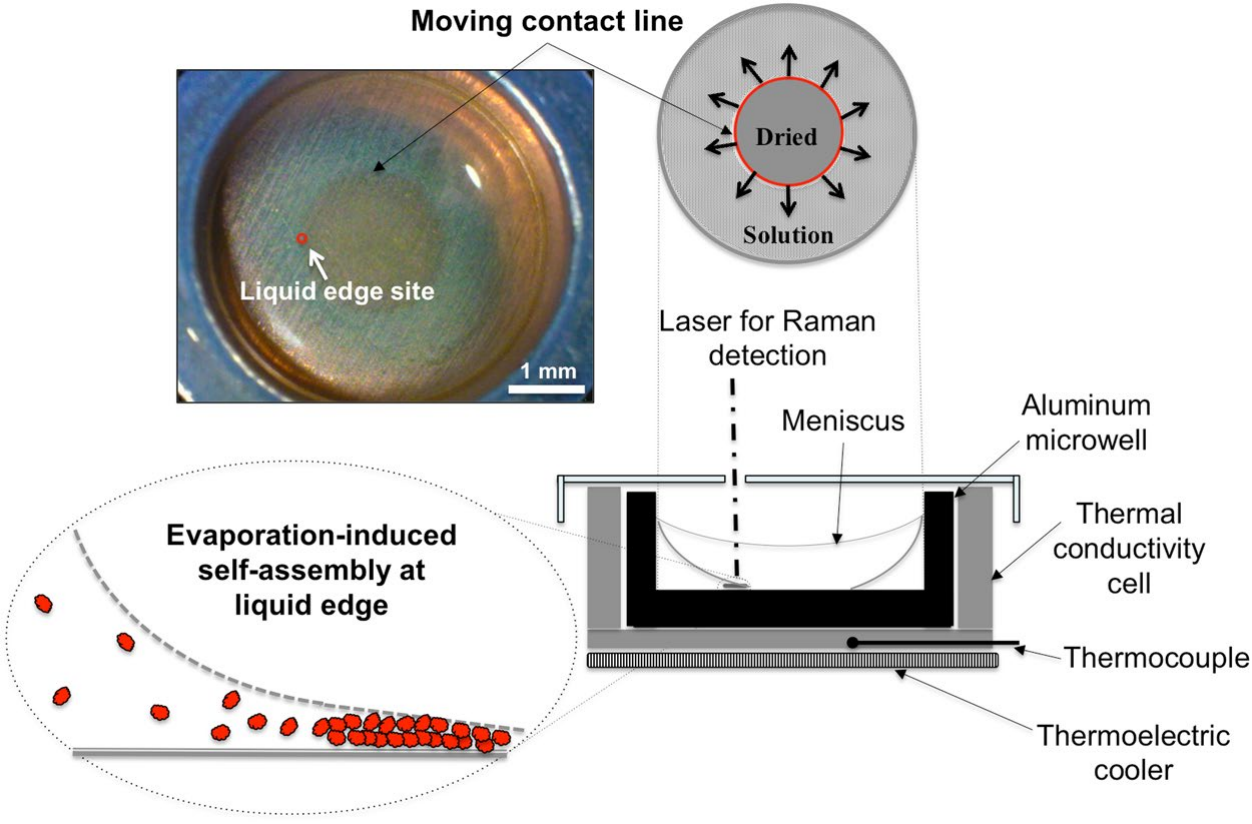
Schematics of the set-up used during the experiments comprising a microwell hosting the protein solution protected by a top cover and inserted in a thermal conductivity cell over a thermoelectric cooler. Raman monitoring of the evaporation-induced assembly of molecules at the liquid edge site is performed by fixing the position for Raman analysis at halfway of the centrifugal movement of the contact line as displayed in the bright field image and relevant sketch on top.

We used a cylinder-shaped 5.5 mm-large and 1.6 mm-high aluminium microwell where a 20 microliter amount of an aqueous solution of protein is easily accommodated by occupying half of the available volume and forming a nearly flat meniscus. The microwell is hosted in a thermal conductivity cell equipped with a top cover and a thermoelectric cooler for lowering the temperature and the evaporation rate when needed, as sketched in Fig. 1 and detailed further in the Materials and Methods section.

**Figure 2:**
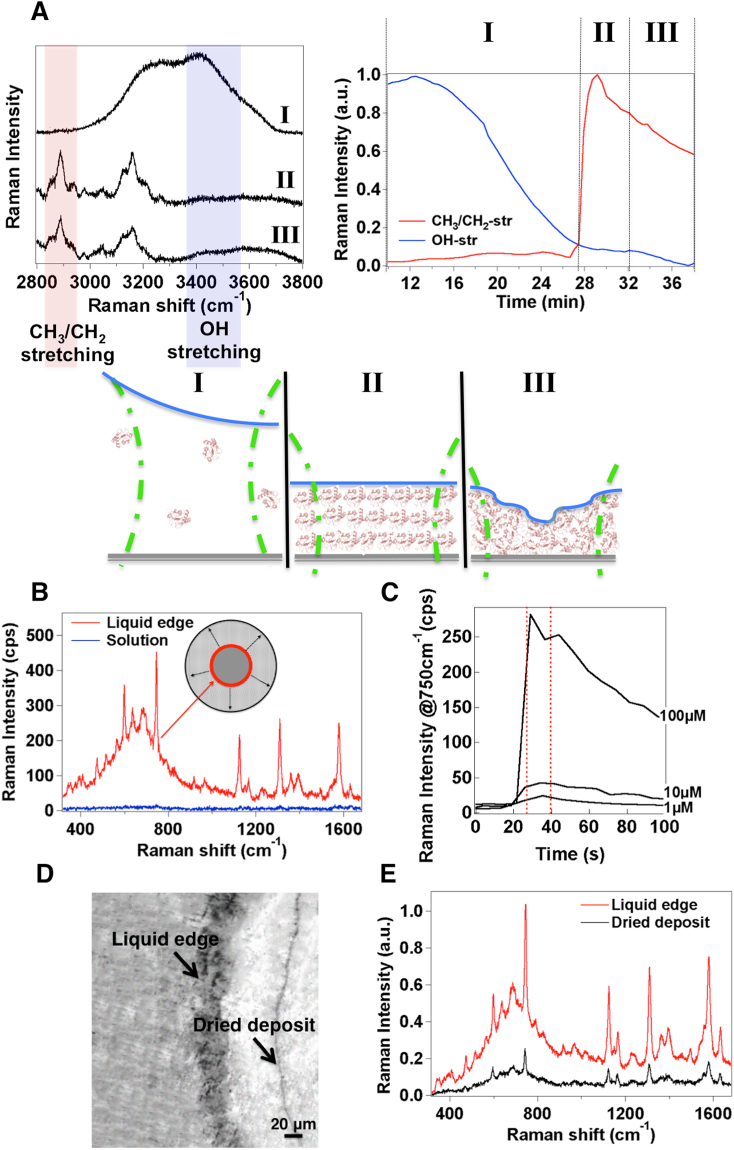
Evidence of a three-stage evaporative process and evaporation-induced Raman enhancement at the liquid edge before formation of desiccated deposits. (**A**) Exemplary Raman spectra of an evaporating cytochrome C (cytC) protein solution including CH_2_/CH_3_- and OH-stretching modes at stages I, II, III (left) and variation in their Raman intensity (right) through these three stages (sketched on the bottom). (**B**) Representative spectrum of 100 μM cytC collected at the liquid edge of the contact line (red). The Raman spectrum of the protein in the solution phase at the same concentration (blue) is also displayed for comparison. (**C**) Variation in Raman intensity *vs* time of the 750 cm^−1^ band of 1, 10 and 100 μM cytC solutions after meniscus rupture, as monitored at the site chosen for Raman analysis shown in Fig. 1. Dashed lines identify those Raman intensity values collected from the liquid edge of the contact line containing assembled molecules (stage II). (**D**) Bright light snapshot of the sample area centered at the liquid edge of a cytC 100 μM solution also showing a deposit ring of desiccated molecules and corresponding Raman spectra (**E**): at the liquid edge site (red) and at the deposit ring site after complete desiccation (black).

During evaporation of the solution at room temperature, its volume is continuously reduced while the meniscus recedes till it ruptures from a continuous state to form a moving contact line. This condition is known as an incomplete meniscus and it produces a circular contact line around the center of the bottom of the microwell (Supplementary Mov. S1), which smoothly moves towards the cylinder walls^22,23^. During the drying process we monitor a fixed point at halfway between the center and the wall of the well bottom (Fig. 1). At this site the Raman profile of the protein is typically collected as a function of the time as exemplified in Supplementary Fig. S1. From this moment on, the drying process of the fluid suspension is noticed to pass through three main stages before complete drying in accordance with previous findings^24,25^: (I) concentration of solute at the moving contact line, (II) formation of a temporary assembly of solute at the edge of the contact line (referred to as “liquid edge” in the followings), and (III) surface adhesion and desiccation onset of the wet assembly. Accordingly, by examining the variation in Raman intensity of the CH_3_/CH_2_- stretching vibrations of the protein from 2860 cm^−1^ to 2970 cm^−1^ (Fig. 2A, Supplementary Fig. S2), the signal, which is initially (stage I) close to our detection limit, undergoes a steepened growth (stage II) followed by a progressive decrease (stage III). Instead, the analysis of the 3350 ÷ 3550 cm^−1^ region of the Raman spectra, where the OH-stretching transitions are located, provides information on the water content in the inspected area of the sample and relative to the protein amount among the different stages. The percentage of water content can be determined as^26^:

1 $$\frac{{I}_{W}}{{I}_{P}}=\frac{{m}_{W}}{{m}_{P}}R$$

2 $$Water\,Content=\frac{{m}_{W}}{{m}_{W}+{m}_{P}}=\frac{{I}_{W}/{I}_{P}}{{I}_{W}/{I}_{P}+R}100$$

where *m*_*W*_ and *m*_*P*_ are water and protein masses in the acquisition volume, *I*_*W*_ and *I*_*P*_ are the integrated Raman signals of water and protein respectively, and *R* is a proportionality constant calculated from the ratio between the Raman signals of water and of a 100 μM protein solution. Water content values of nearly 100% at the onset of the drying process (stage I) were progressively reduced down to values below 10% upon complete drying of the solution (i.e. desiccation at the completion of the stage III). During the intermediate stage (II), water content values between 30% and 40% were typically calculated, indicating that protein molecules are still fully hydrated in accordance with previous literature^27^. At this stage, an increase in protein concentration due to molecular confinement causes protein-protein interactions and self-assembly via hydrogen bonds and van der Waals forces between the hydrophobic parts of the protein. Hydration is beneficial in assisting this process by providing enough flexibility for molecular rearrangement and packing^28,29^. In this crowded environment, slight changes in the hydrophobic residues of the molecules without any specific modifications to secondary and tertiary structures of the protein have been previously reported^27,30^.

Systematic experiments have been then carried out on cytochrome c (cytC), which represents a popular model protein well-characterized over several Raman studies (Supplementary Table S1). We verified that an immediate consequence of the evaporation-driven accumulation of protein molecules at the liquid edge is a 50-fold minimum increase in Raman intensity of their vibrational modes as compared to the solution phase (Fig. 2B). Thus a Raman analysis focused on inspecting the contact line of the liquid shall greatly benefit from an improved limit of detection on account of the high molecular density temporarily established there (see Fig. 2C).

Upon complete drying of the solution, a macroscopic precipitation pattern in the form of concentric rings of protein deposits is detected by moving along the radius of the microwell (Fig. 2D and Supplementary Fig. S3) and in line with previous observations^21,31^. A comparison between the Raman profiles of the protein collected at the liquid edge and within ring deposits (Fig. 2E, Supplementary Fig. S3) reveals sharper peaks and improved spectrum details at the former site. Conversely, spectral broadening with reduced spectral information as observed after drying can be ascribed to molecular degradation, as reported by several studies on nonnative conformational protein transitions^32,33^. The absence of these features at the liquid edge suggests that the protein molecules in the assembled phase maintain their natural state, which is favoured by the wet environment mostly retained there (Fig. 2A).

### Control over the evaporative dynamics for protein accumulation at the liquid edge

Our following efforts have been spent in defining the experimental conditions to optimize the detection method and to further improve the Raman signal of the protein at the liquid edge site. The evaporation process of the protein solution occurring in the microwell is governed by recurrent stick-slip behaviour of the contact line. At the beginning the contact line motion is expected to drag the protein molecules with it, pulling them toward the walls of the well. With an increase in the concentration of the solution, the contact line is pinned at one location for a while, which triggers gathering of molecules in temporary domains at the rim of the contact line followed by deposition of a part of them on the bottom of the well. Then the contact line moves apart from the line of deposited solute (depinning) bringing together the rest of the molecules and is repinned at a consecutive location and so forth. Pinning and depinning can occur once, twice, or many times, depending on substrate, molecule type, and evaporation conditions^34^.

**Figure 3:**
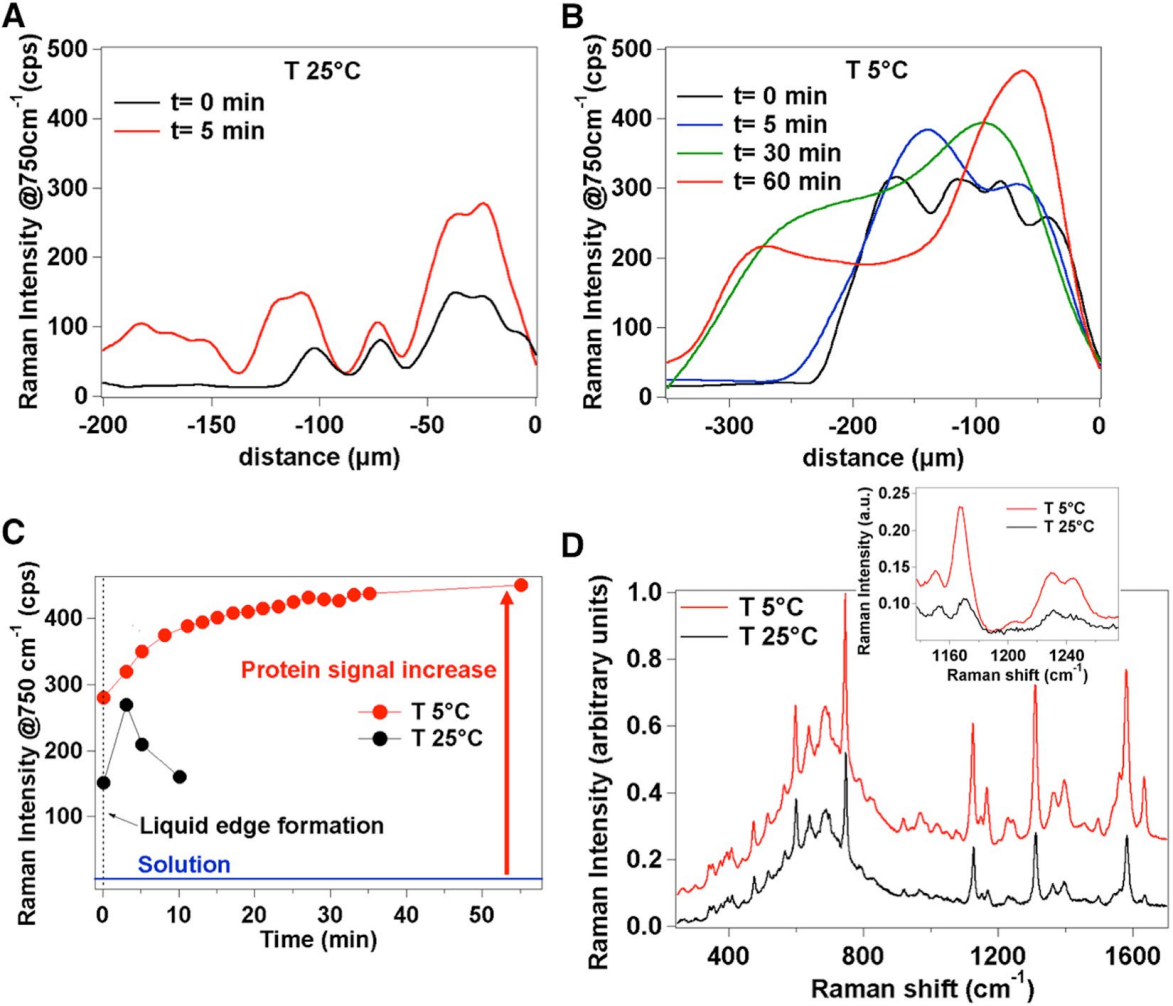
Different regimes of protein accumulation at liquid edge obtained by slowing down the evaporation kinetics. Variation in Raman intensity of the 750 cm^−1^ signal of 100 μM cytC as obtained by radial Raman mapping (15 μm stepped) from the liquid edge position (0 μm) toward the solution phase at 25 °C (**A**) and 5 °C (**B**). Measurements are repeated at different times after the formation of the first liquid edge phase followed by adjusting the temperature at 25 °C or at 5 °C (defined as *t = *0). (**C**) Raman intensity of the 750 cm^−1^ signal at the liquid edge monitored by repeated acquisitions over a total of 10 min at T = 25 °C and of 60 min at T = 5 °C. Values represent the average of 3 random points of the liquid edge from 3 replicates. The relative standard deviation (RSD) on Raman intensity is 5%. (**D**) Comparison between Raman profiles of 100 μM cytC at the liquid edge site collected during 60 min at 5 °C and averaged over 165 accumulations (red line) and during 5 min at 25 °C and averaged over 10 accumulations (black line). Inset: enlargement of the 1100–1280 cm^−1^ spectral region to highlight the intensity and quality differences between the two profiles. See Supplementary Fig. S5 for an analogous comparison on HSA.

In recent literature, a kinetic control over the evaporation and deposition regime from a sessile drop of a solution was achieved by increasing the temperature and thus the evaporation rate of the system^35^. At high temperature, the increased evaporation rate prevents the solute from being transported toward the drop edge producing a uniform deposition on the surface. In our experiments we were aimed at obtaining an opposite result i.e. by decreasing the evaporation rate, the pinning state lasts for a longer time and molecular accumulation at the liquid edge site can be encouraged, ultimately increasing the amplitude of the temporary domains and extending the time for collecting repeated spectra. According to this picture, we tracked the evaporative kinetics of the protein solution at decreasing temperature values by cooling down the microwell. To this aim, after meniscus rupture and subsequent formation of the first liquid edge phase at room temperature, a mapping along the direction of the moving contact line is carried out at different times once the temperature is adjusted to a fixed value within the 25 °C ÷ 5 °C range (defined as *t* = 0) by means of the thermoelectric cell. After only 5 min at T = 25 °C, new pinned positions apart from the moving contact line where molecules form assemblies start appearing (Fig. 3A), in accordance with a rapid evaporation process and multiple depositions of protein. Conversely, by decreasing the temperature below 15 °C, protein accumulation in the first pinning site is increased and the formation of a new assembly is not observed before 30 min (not shown). At 5 °C the molecular accumulation at the liquid edge is even more effective and the formation of the second assembly is dramatically postponed (Fig. 3B). At this temperature, the system remains in a quasi-frozen state for about 60 min with the contact line nearly fixed in the initial pinning position. The low temperature alters the stick-slip movement of the contact line so that each ‘sticking’ interval increases while molecules keep accumulating in the liquid edge region. As a matter of fact, the Raman signal collected over 60 min upon the formation of the pinned contact line at T = 5 °C reaches about 2-fold higher intensity values (Fig. 3C) as compared with that collected over merely 5 min of available time at 25 °C before desiccation onset providing an overall 100-fold increase relative to the initial solution phase (Fig. 3C). The choice of an experimental setting for slowing down the evaporative process turned out also favourable in improving the signal-to-noise ratio in turn offering additional sub-structural features throughout the protein profile (Supplementary Fig. S4). This is exemplified in Fig. 3D where the protein spectrum resulting from averaging 165 separate measurements recorded during 60 minutes of liquid edge life time at 5 °C is compared with the corresponding from 10 measurements performed within 5 minutes at 25 °C. Finally, we note that after complete desiccation of the protein solution at 5 °C, the signal undergoes an approximate 5-fold drop in intensity and loosening of spectral detail when evaluated within the dried ring deposits formed on the surface of the microwell (Supplementary Fig. S3) confirming a tendency previously observed at 25 °C (Fig. 2E).

### Surface-enhanced Raman (SER) detection by addition of a plasmonic coating

Once the most convenient experimental parameters for promoting molecular assembly at the liquid edge have been established, we evaluated the benefit of adding a SER-active interface in order to further boosting the Raman signal. Specifically, Raman measurements were repeated upon coating^36,37^ the bottom of the microwell with a dispersion of 50 nm-size silver nanoparticles with a cubic shape (silver nanocubes, AgNCs)^38^ (Supplementary Fig. S6). These represent a convenient choice in SERS owing to intense electromagnetic field localized at their corners and their ability to produce 2-dimensional layers with intensified gap regions between closely spaced nanoparticles^39–41^. After coating, the surface plasmon peak at 410 nm that is typical of isolated AgNCs in solution is replaced by a broader peak at 580 nm corresponding to assembled nanoparticles (Supplementary Fig. S6), which well matches our laser excitation conditions^42^.

**Figure 4:**
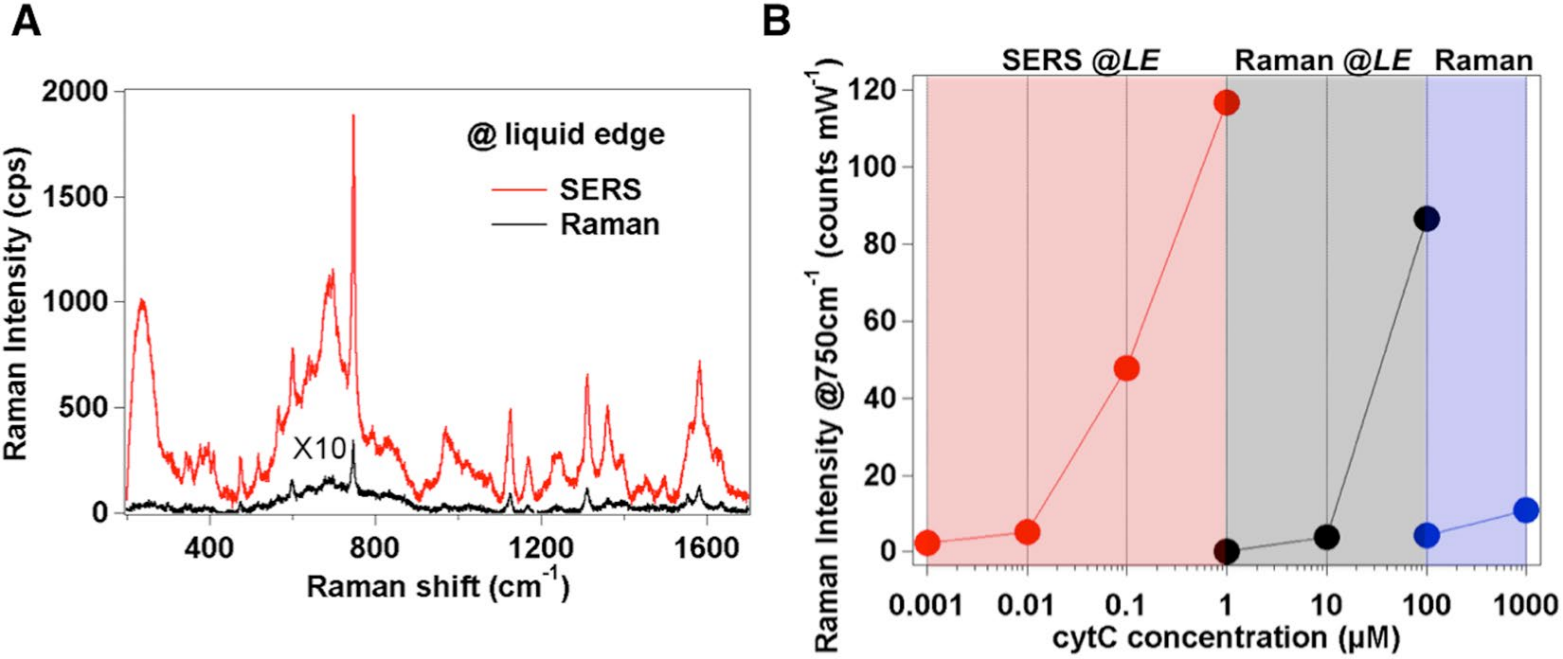
SERS effect at the liquid edge. (**A**) Raman spectra of 1 μM cytC at the liquid edge after the addition of a AgNC coating (red) or without it (black). (**B**) Plot of the 750 cm^−1^ band intensity *vs* concentration of cytC in the sub-micromolar range as measured at the liquid edge site in the SERS (SERS @LE) and Raman (Raman @LE) setup or collected from the bulk solution (Raman). Raman intensity values (average of 3 random points of the liquid edge from 3 replicates for Raman @LE and SERS @LE) are normalized for the power and integration time. RSD lies in the 5% ÷ 10% range.

We verified that the presence of the SERS-active coating is able to supply the system with an additional 100-fold amplification in the Raman spectra on average (see Fig. 4A). Afterwards, a systematic analysis during the evaporation of cytC solutions at decreasing concentration values was performed to establish a limit of detection of the improved assay. We were able to detect a protein signal localized at the liquid edge down to submicromolar concentration with a regular scaling in the 1 × 10^−6^ M ÷ 1 × 10^−9^ M range (Supplementary Fig. S7), overcoming previous outcomes resulting from the analysis of dried drops on both naked^10,13^ or nanostructured-plasmonic^43,44^ substrates. Furthermore, the molecular undersaturation of the hot-spot sites on the nanoparticle surface supplies the system with potential for quantitative analyses within an extended concentration range.

In Fig. 4B a summary of the Raman measurements obtained with the use of the optimized system at the liquid edge or from bulk solution over a wide protein concentration range clearly evidences the benefits offered by using the proposed optofluidic approach. A quantitative estimation of the overall signal amplification can be provided by the analytical enhancement factor or SERS gain *G*. *G* is defined as the ratio between SERS (*I*_*SERS*_) and Raman (*I*_*Raman*_) intensities normalized for concentration (*C*), integration time (*t*) and power (*P*) values employed during the SERS and Raman measurements while experimental parameters such as laser wavelength, microscope objective and grating are maintained identical^45,46^:

3 $$G=\frac{{I}_{SERS}/(t\,\times \,P\,\times {C}_{SERS})}{{I}_{RAMAN}/(t\,\times \,P\,\times \,{C}_{RAMAN})}$$

By considering the 750 cm^−1^ band intensity in the SERS spectrum of a 10^−9^ M cytC solution with the analogue of the Raman spectrum of a 10^−4^ M solution (Supplementary Fig. S8), we calculated a *G* ≈ 2 × 10^5^. This result is in line with the signal enhancement provided by recently introduced and most powerful SERS assays for biomolecule detection based on aqueous dispersions of isolated^47^ or aggregated^48,49^ plasmonic nanoparticles. Remarkably, the 2D geometry of the optofluidic platform holds the promise for an optimal control over the Raman measurement with minimal signal fluctuations and low RSD values (see Fig. 4B), which instead is critical when operating in 3D liquid spaces.

## Conclusions

An aqueous solution of protein is allowed to evaporate on the bottom of a circular microwell equipped with a thermoelectric cooling cell for fine-tuning of the evaporation rate at fixed temperature values. The radial convective flows during evaporation carry molecules from the center toward the microwell walls inducing a progressive gathering of molecules at the edge of the evaporating liquid. Here a significant amplification of the Raman signal is produced, which can ultimately reach a factor of 10^5^ when the evaporation rate is lowered to below room temperature values and the microwell is coated with a plasmon nanoparticle layer. Importantly, the assembled proteins at the liquid edge maintain a native-like state without displaying spectral changes that instead are observed upon complete removal of water molecules and desiccation.

The combined microfluidic/Raman system based on evaporation-induced molecular confinement at the liquid edge represents a novel approach for effective detection and characterization of sub-micromolar quantities of biomolecules in wet conditions.

## Materials and Methods

### Evaporation in the microwell reservoir and Raman monitoring of the process

A volume of 20 μl of an aqueous protein solution is added with a micropipette to an aluminum microwell (40-μL volume capacity). The microwell is precisely accommodated in an aluminum block sample holder equipped with thermoelectric cooler cell (Thorlabs, TEC 3-2.5) for temperature cooling. The aluminum block is built to fit in the sample holder of the microRaman stage used for the measurements. Different concentrations of protein solutions in ultrapure MilliQ water were evaporated in the microwell under temperature control.

The synthesis of AgNCs was made by following a published protocol and fabrication details are reported in SI. A 40-μL aliquot of nanoparticle solution is gently dropped into the microwell and then left undisturbed overnight in normal laboratory conditions at room temperature. Aftherwards, a plasma pretreatment (Diener ZEPTO operated at 40 kHz, 90 W and 1 mbar in air) for 10 min is performed just before adding the protein solution for the Raman measurements.

### Raman and SERS experiments

Raman and SERS measurements were carried out using a micro-Horiba Xplora system coupled to a 532 nm wavelength laser. The backscattered light was collected by a 10 × microscope objective with 0.2 NA, which generates a ~7-μm large laser beam waist. Integration times of 1 to 5 s, laser power values in the 2 mW (SERS measurements) ÷ 5 mW (Raman measurements) range and a grating of 1800 cm^−1^ were employed. Raman mapping measurements *vs* time (Fig. 2A,C) and distance (Fig. 3A,B) were acquired with a step of 10 s and 15 μm, respectively.

## Electronic supplementary material

Supplementary Information

Supplementary Mov. S1
