# Supplementary material for: Triggering molecular assembly at the mesoscale for advanced Raman detection of proteins in liquid
Source: Sci Rep. 2018 Jan 18;8:1033. doi: 10.1038/s41598-018-19558-w (PMC5773671; doi:10.1038/s41598-018-19558-w)
Supplement: Supplementary file 1 — Supplementary Information [file 41598_2018_19558_MOESM1_ESM.pdf]

Supplementary Information

# Triggering molecular assembly at the mesoscale for advanced Raman detection of proteins in liquid

*Martina Banchelli*[a], *Marella de Angelis*[a], *Cristiano D'Andrea*[a], *Roberto Pini*[a], *and Paolo Matteini*[a*]

[a] Institute of Applied Physics 'Nello Carrara' of the National Research Council (IFAC-CNR), via Madonna del Piano 10, Sesto Fiorentino, Florence (Italy)

*corresponding author:* p.matteini@ifac.cnr.it

## Raman spectra of proteins during different evaporation stages

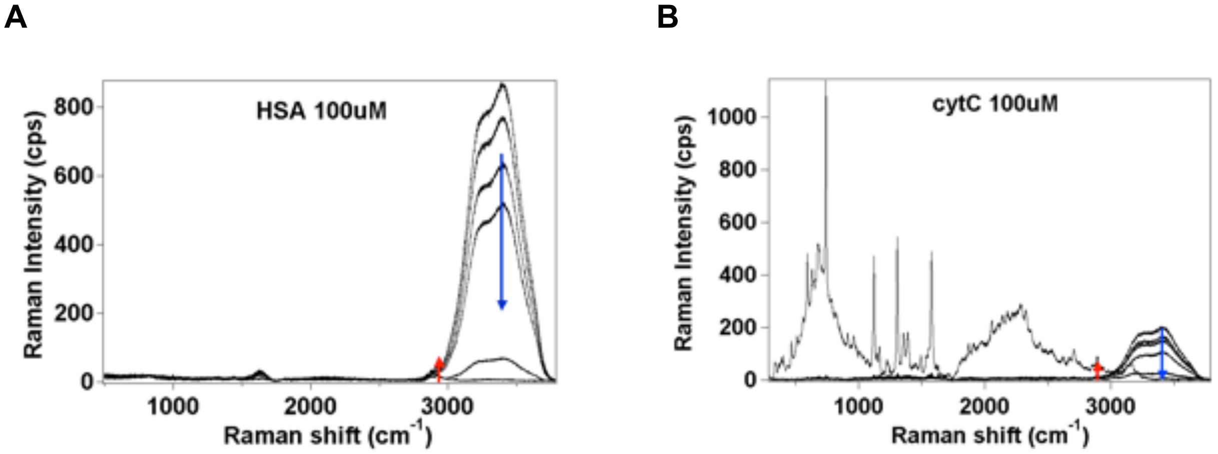


**S1.** Raman spectra acquired at halfway between the center and the wall of the microwell during the evaporation of 100 μM human serum albumin (HSA) (**A**) and cytochrome C (cytC) (**B**) solutions.

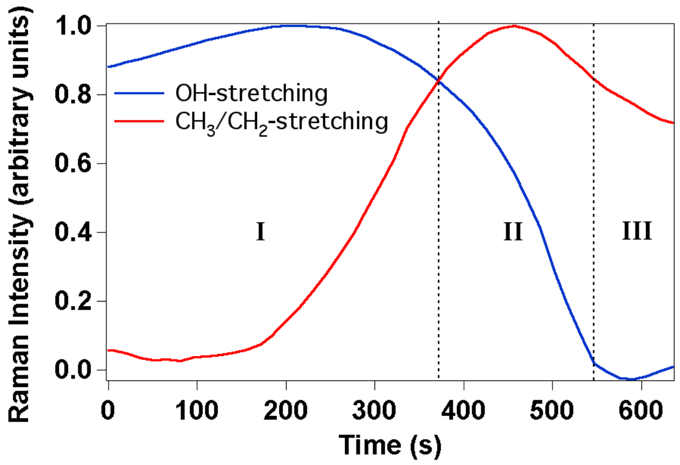


**S2.** Three-stages evaporative process and evaporation-induced Raman enhancement at the liquid edge of a HSA solution. Variations in the Raman intensities of the OH-stretching vibrations (integrated signals in the 3350-3550 cm$^{-1}$ range) and of the $CH_3$/$CH_2$- stretching vibrations (integrated signals in the 2860 -2970 cm$^{-1}$ range) of 100 μM HSA during stages I, II and III.

**Table S1. Assignment of the main Raman peaks of cytC[1,2]**

| Frequency ($cm^{-1}$) | Assignment |
|---|---|
| 690 | ν(pyr deform)$_{sym}$ |
| 750 | ν(pyr breathing) |
| 920 | δ(pyr deform)$_{asym}$ |
| 1130 | ν(pyr half-ring)$_{sym}$ |
| 1170 | ν(pyr half-ring)$_{sym}$ |
| 1230 | δ($C_m$-H) |
| 1311 | δ($C_m$-H) |
| 1364 | ν(pyr half-ring)$_{sym}$ |
| 1397 | ν(pyr quarter-ring) |
| 1497 | ν($C_\alpha$-$C_m$)$_{sym}$ |
| 1545 | ν($C_\beta$- $C_\beta$) |
| 1585 | ν($C_\alpha$-$C_m$)$_{asym}$ |
| 1634 | ν($C_\alpha$-$C_m$)$_{asym}$ |

**Multiring pattern deposition after evaporation of a cytC solution inside the microwell. Effects of evaporation-rate control.**

The first systematic attempt to develop a quantitative theory to predict which ring pattern shall result from pinning and depinning dynamics was recently made by Frastia et al.[3]. Their model is based on a lubrication equation for hydrodynamics and a mass balance equation for the solute concentration. During the first part of the process, when the concentration is low, the convection is rapid and no pinning can occur. Then the concentration of molecules increases as evaporation is underway, leading to a rise in viscosity, and the suspension may become hydrodynamically jammed forming a pinned line of solute. Eventually the fluid behind this line retreats from it and additional lines might form. A variety of scenarios are possible at this stage, as controlled by concentration, evaporation rate, surface tension and fluid density as described in ref. 3.

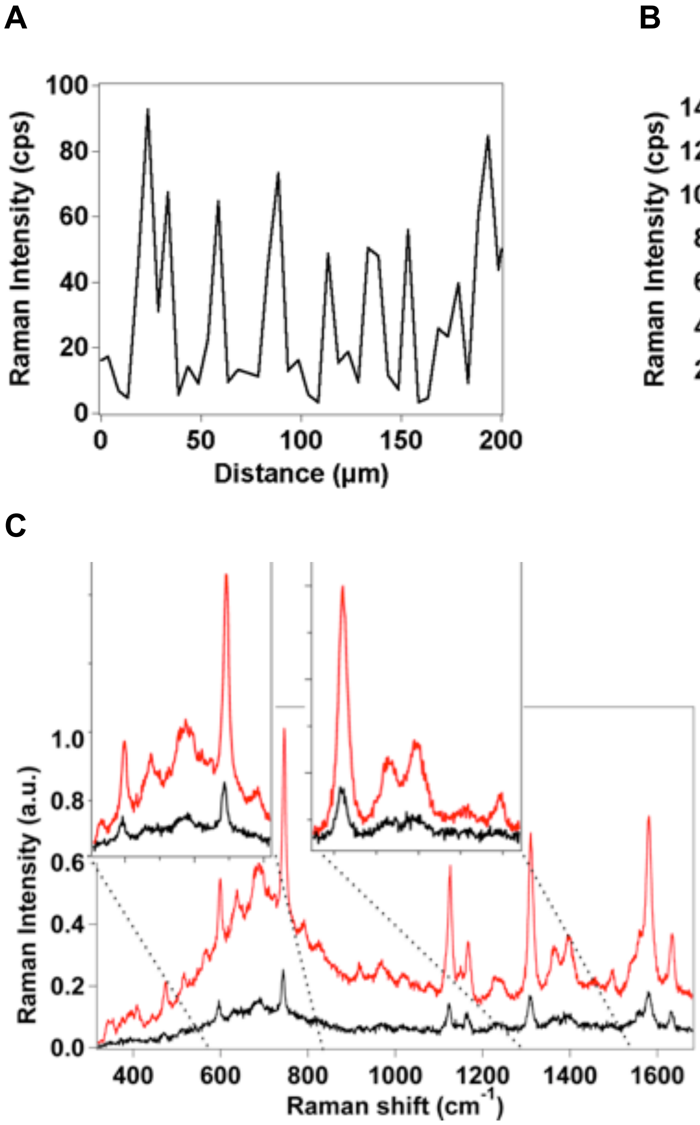


**S3.** Variation in Raman intensity of the 750 $cm^{-1}$ signal of a 100 μM cytC solution after drying at T = 25°C (**A**) and at T = 5°C (**B**) as obtained by radial Raman mapping (15 μm stepped) from the center (0 μm) toward the walls of the microwell. (**C**) Selected regions of the Raman spectra of a 100 μM cytC solution collected at the liquid edge (red line) and after drying (black line)

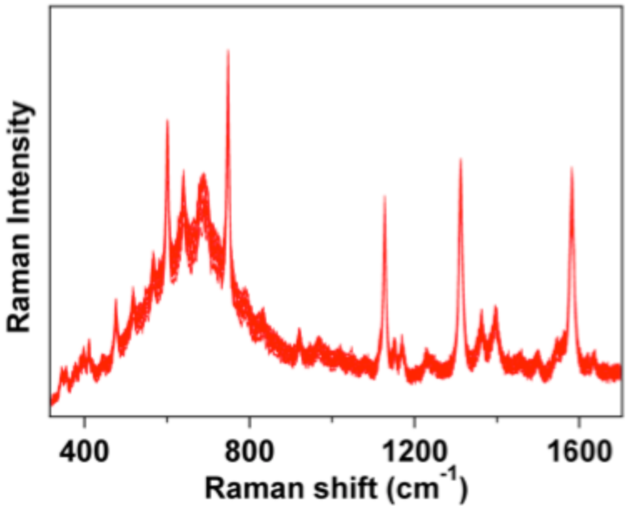


**S4.** Series of 10 sequential spectra of a 100 µM cytC solution at the liquid edge at 5°C. The spectra were collected with a 3 sec integration time and spaced by 70 sec from one another.

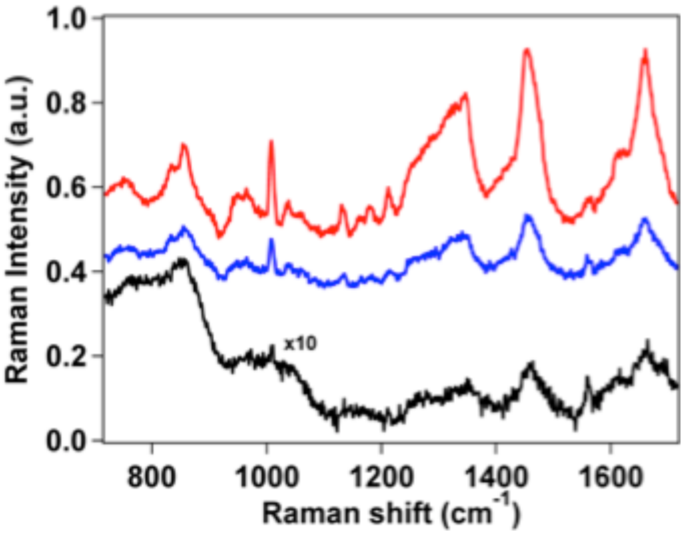


**S5.** Raman profiles of 100 µM HSA collected at the liquid edge site during 60 min at 5°C and averaged over 165 accumulations (red line) and during 5 min at 25°C and averaged over 10 accumulations (blue line) compared with the Raman spectrum of a 100 µM HSA solution (black line).

**Fabrication and characterization of the AgNCs**

Ethylene glycol was purchased by Schaefer while all the other reagents were purchased by Sigma-Aldrich and used without any further purification. 10 ml of ethylene glycol were placed into a flask and heated under magnetic stirring in an oil bath at 150°C for 1 hour in a nitrogen flow. 0.175 ml of

0.72 mg/ml sodium sulfide solution and 3.75 ml of 20 mg/ml polyvinylpyrrolidone solution were subsequently added to the flask. 1.25 ml of a silver nitrate solution (48 mg/ml) was added dropwise into the reaction flask. The reaction was stopped after 40 min in an ice-bath by adding 30 ml of acetone. Nanoparticles were centrifuged at 10000 g for 30 min and disperesed in ethanol using an ultrasonic bath. The washing procedure was repeated three times to remove all the reagents. The yield of the reaction was around 80%. The suspension of AgNCs was finally stored at -18°C.[4]

An AgNC concentration of 1.74 × $10^{-10}$ M (1.05 × $10^{11}$ AgNCs/mL) after redispersion was calculated by measuring the extinction of the colloidal solution measured at 430 nm in a 1 cm-side cuvette and by considering a value of 5.5 × $10^{-10}$ $M^{-1}$ $cm^{-1}$ as the extinction coefficient of Ag[5].

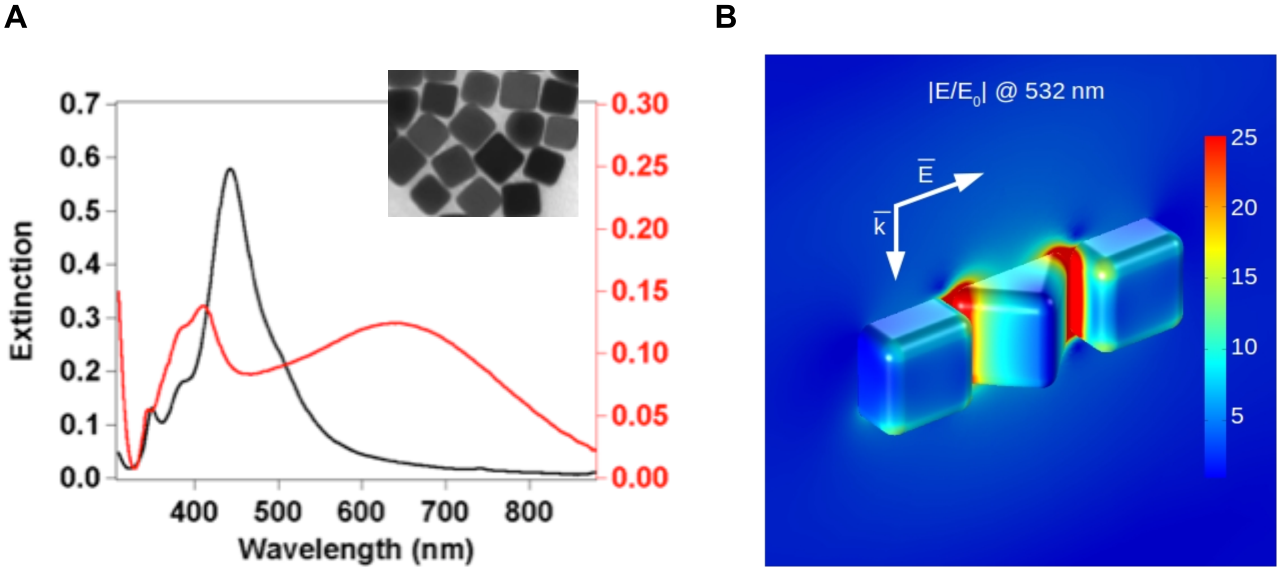


**S6.** (**A**) Extinction spectrum of as-synthesized AgNC dispersion in ethanol (black) and of an assembled layer of AgNCs upon evaporation of 1 μL of concentrated solution on quartz (red). Inset: TEM image of the as-synthesized AgNCs. (**B**) FEM simulation of the spatial distribution of the E-field for a 3-cube assembly in aqueous medium (cubes of 50 nm side length and with a corner radius of 8 nm were considered). A slice crossing the cubes displays the E-field distribution in the proximity of the assembly.

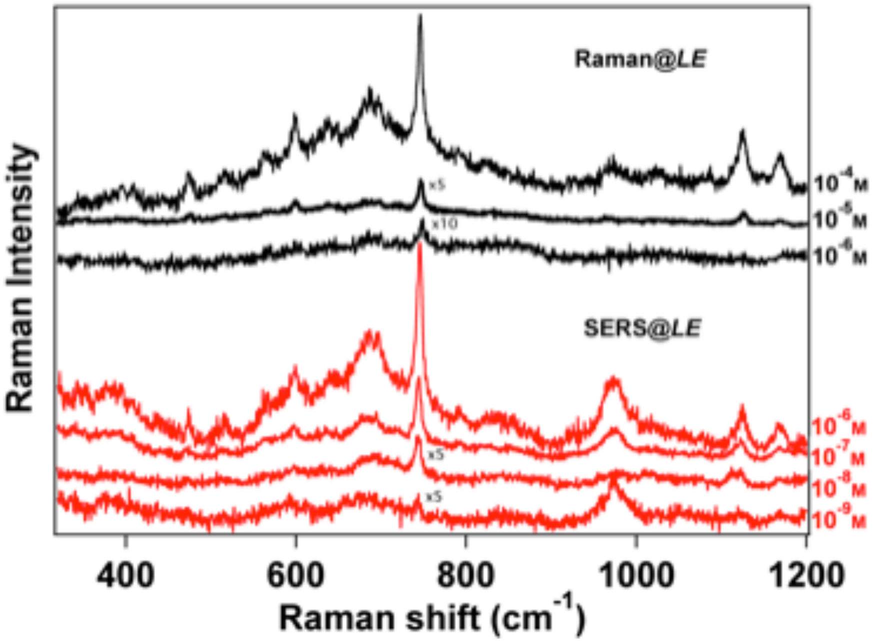


**S7.** Raman and SERS spectra of cytC at the liquid edge at different concentration values.

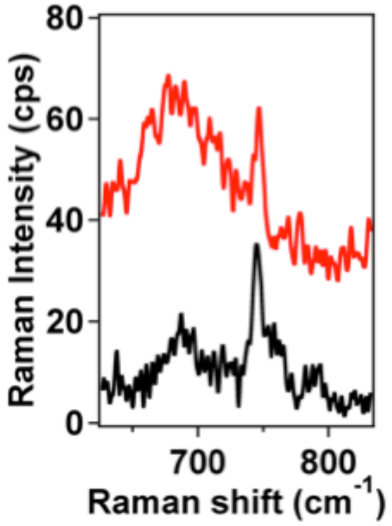


**S8.** Comparison between the 750 cm$^{-1}$ Raman ($10^{-4}$ M aqueous solution, black line) and SERS ($10^{-9}$ M liquid edge, red line) bands of cytC.

## References


1 Dick, L.A., Haes, A.J. & Van Duyne, R.P. Distance and orientation dependence of heterogeneous electronic transfer: a surface-enhanced resonance Raman scattering study of cytochrome c bound to carboxylic acid terminated alkanethiols adsorbed on silver electrodes. *J. Phys. Chem. B*, **104**, 11752-11762 (2000).

2 Delfino, I, Bizzarri, A.R. & Cannistraro, S. Single-molecule detection of yeast cytochrome c by surface-enhanced Raman spectroscopy. *Biophys. Chem.* **113**, 41-51 (2005).

3 Frastia, L., Archer, A. J. & Thiele, U. Dynamical model for the formation of patterned deposits at receding contact lines. *Phys. Rev. Lett.* **106**, 106, 077801 (2011).

4 Banchelli, M. *et al.* Controlled graphene oxide assembly on silver nanocube monolayers for SERS detection: dependence on nanocube packing procedure. *Beilstein J. Nanotechnol.* **7**, 9-21 (2016).

5 Lu, F. *et al.* Discrete nanocubes as plasmonic reporters of molecular chirality. *Nano Lett.* **13**, 3145-3151 (2013).
